# ASSOCIATION OF HAVING DOMESTIC WORKERS WITH ELDERLY’S SOCIAL PARTICIPATION, VOLUNTEERING, AND LIFELONG LEARNING

**DOI:** 10.1093/geroni/igae098.4332

**Published:** 2024-12-31

**Authors:** Youjuan Zhang, Xue Bai

**Affiliations:** The Polytechnic University, Hong Kong, Hong Kong; The Polytechnic University, Hong Kong, Hong Kong

## Abstract

Due to the family caregiver shortage and life expectancy increase, there has been an emerging worldwide trend of migrant domestic workers (MDWs) providing elderly care. However, the association of having MDWs at home with older adults’ psychosocial wellbeing has been under investigated. This study used data from the Panel Study of Active Ageing and Society (PAAS, conducted from June to November 2022) among 5007 Cantonese-speaking residents aged ≥50 years (64.2 ± 8.4 years, women: 53.5%) in Hong Kong. Participants self-rated their social participation frequency on a 20-item four-point scale, from “1.=never” to “4.=almost every day”. Moreover, participants reported their volunteering experience by rating their monthly volunteering time (from “0.=none” to “5.≥20 hours”), and their learning activities over the past two years using a simple yes/no response. On average, participants scored 29.32 (SD: 5.01) out of 80 on social participation, 17.6% engaged in volunteering, and 4.4% had learning activities. After adjusting age, sex, living status, education level, family income, and physical status, results from linear and logistic regression models showed that having an MDW at home was associated with enhanced social participation (β=1.24, p< 0.001), and increased odds of participating in volunteering (OR=1.38, 95% CI: 1.05-1.81, p=0.02) and learning activities (OR=2.38, 95% CI: 1.60-3.53, p< 0.001) among older adults. Social participation, volunteering, and lifelong learning are crucial for fostering community engagement and quality of life. Our findings imply that integrating MDWs into elderly care not only supports older adults’ psychosocial well-being but also could contribute to community development as a whole.

